# Mortality Rate of Lymphoma in China, 2013–2020

**DOI:** 10.3389/fonc.2022.902643

**Published:** 2022-06-07

**Authors:** Weiping Liu, Jinlei Qi, Jiangmei Liu, Yuqin Song, Lijun Wang, Maigeng Zhou, Jun Ma, Jun Zhu

**Affiliations:** ^1^ Key Laboratory of Carcinogenesis and Translational Research (Ministry of Education), Department of Lymphoma, Peking University Cancer Hospital and Institute, Beijing, China; ^2^ National Center for Chronic and Noncommunicable Disease Control and Prevention, Chinese Center for Disease Control and Prevention, Beijing, China; ^3^ Department of Hematology & Oncology, Harbin Institute of Hematology & Oncology, Harbin, China

**Keywords:** lymphoma, Hodgkin disease, non-Hodgkin lymphoma, epidemiology, mortality

## Abstract

Lymphoma is a malignant disease that threatens human health and imposes a significant burden on the society burden; however, there are limited accurate mortality data on lymphoma in China. The present study aimed to analyse lymphoma-associated mortality at the national and provincial levels in mainland China. Mortality data of lymphoma was extracted from the disease surveillance system of the Chinese Center for Disease Control and Prevention. Mortality was represented by the number of deaths, crude mortality rate, and age-standardized mortality rate. Temporal trends in mortality rates were examined using the fitting joinpoint models. Lymphoma accounted for 31,225 deaths in 2020, of which 1,838 and 29,387 were due to Hodgkin lymphoma (HL) and non-Hodgkin lymphoma (NHL), respectively. The age-standardized mortality rate per 100,000 population was 1.76 for lymphoma, 0.10 for HL, and 1.66 for NHL. The mortality rate increased with age, reaching a peak in the age group of 80–84 years for HL and over 85 years for NHL. Moreover, the death risk due to lymphoma was approximately 1.5–2 times greater in males than in females in all age groups. The mortality rate was higher in eastern China than in central and western China, indicating a heterogeneous distribution at the provincial level. During 2013–2020, the mortality rate of lymphoma decreased by 1.85% (−22.94% for HL and −0.14% for NHL). In conclusion, the mortality of lymphoma varied by sex, age, and regions, which highlighted the need of establish differentiated strategy for disease control and prevention.

## Introduction

Lymphoma is a malignant disease that threatens human health. A systematic analysis based on the GLOBOCAN 2020 study from the International Agency for Research on Cancer revealed 83,087 incident cases and 23,376 deaths due to Hodgkin lymphoma (HL) and 544,352 incident cases and 259,793 deaths due to non-Hodgkin lymphoma (NHL), which accounted for 3.2% of all cancer cases and 2.8% of all cancer deaths worldwide (1). Compared with the results from the GLOBOCAN 2018 study, the incident cases increased by 3.9% and deaths decreased by 10.7% for HL, whereas the incident case and deaths increased by 6.8% and 4.5% for NHL, respectively (2).

China has a lower burden of lymphoma than the western countries. For example, there were an estimated 136,960 new cases of lymphoid malignancy with an age-standardized incidence rate of 34.4 per 100,000 population in the United States in 2016 (3). The new cases and age-standardized incidence rate per 100,000 population were 8,500 and 2.7 for HL, and 125,850 and 31.1 for NHL, respectively (3). During the same period, there were an estimated 6,900 and 68,500 incident cases with an age-standardized incidence rate of 0.46 and 4.29 per 100,000 population for HL and NHL in China (4). Notably, the mortality rate of lymphoma and myeloma showed a significant upward trend with an annual increase of 4.5% from 2004 to 2016 (5). In 2017, the incidence and mortality rates of NHL ranked 14th 12th, while the incidence and mortality rates of HL ranked 31st among all cancers in China (6).

Due to the low incidence rate of HL, NHL, and multiple myeloma, these three diseases are commonly grouped into the same classification in China. For example, the National Central Cancer Registry of China estimated that there were 52,100 deaths due to lymphoma and myeloma in 2015, but the accurate mortality data of lymphoma alone were not determined (7). In the current study, we conducted a comprehensive analysis of lymphoma-associated mortality at the national and provincial levels in mainland China.

## Methods

The study was approved by the Ethics Committee of the Chinese Center for Disease Control and Prevention (Beijing, China).

### Data Sources

Mortality data were collected from the Chinese Center for Disease Control and Prevention–Disease Surveillance Points (CDC–DSP) system. This system consists of 605 surveillance points and covers a population of 323.8 million (24.3% of the total population of the country) across 31 provinces.

National age-specific population data were obtained from the National Bureau of Statistics of China (http://data.stats.gov.cn). The 2010 census population data of China were used to determine the age-standardized mortality rates by Chinese standard population (ASMRC). The Segi’s population was used to calculate the age-standardized mortality rate worldwide (ASMRW) (8).

### Data Collection

The daily death records from 1 January 2013 to 31 December 2020 were collected from the CDC-DSP system. International Classification of Diseases- 10 codes were used to identify HL (C81–C81.99) and NHL (C82–C86.6, C96–C96.9).

### Quality Control

The quality control procedures for CDC-DSP system includes annual training of standard work flow, random checking of the accuracy of disease classification and duplication, which is done at the county, province, and national levels. The underreporting rate was evaluated by retrospective survey every 3 years, which was 9.4% in the recent period of 2015−2017.

### Classification

The geographic unit includes 31 provinces, municipalities and autonomous regions in mainland China, which was referred to as provinces in the present study. The geographic area was divided into eastern China (including Beijing, Tianjin, Hebei, Liaoning, Shanghai, Jiangsu, Zhejiang, Fujian, Shandong, Guangdong and Hainan), central China (including Shanxi, Jilin, Heilongjiang, Anhui, Jiangxi, Henan, Hubei and Hunan), western China (including Inner Mongolia, Guangxi, Chongqing, Sichuan, Guizhou, Yunnan, Tibet, Shaanxi, Gansu, Qinghai, Ningxia and Xinjiang) (9). Urban/rural classification was made according to administrative characteristics (county as rural and district in cities as urban) (10).

### Statistical Analysis

The mortality rates were calculated by the method of the following formula: estimated mortality rates = reported mortality rates/(1 - underreporting rates). The estimated deaths due to lymphoma were generated by the sum of the products of the age-specific mortality rates and the corresponding population in each stratum. Temporal trends in mortality rates from 2013 to 2020 were examined by fitting joinpoint models (version 4.6.0.0; National Cancer Institute). Changes were represented by the average annual percent change (AAPC) and their corresponding 95% confidence interval (CI) over the entire period. The term “increase” or “decrease” was used to describe the trends when the slope was statistically significant. For nonstatistically significant trends, the term “stable” was used.

The changes in the number of deaths between 2013 and 2020 was attributed to population growth, population structure, and age-specific mortality rate. The decomposition analysis used two counterfactual scenarios to calculate the number of deaths. The first scenario assumed that the total population grew but the population structure and age-specific mortality rate remained unchanged from 2013 to 2020. The difference between the number of deaths observed in 2013 and the first scenario was the change in the number of deaths exclusively attributable to population growth. The second scenario assumed that the total population grew and the population structure changed, but the age-specific mortality rate remained unchanged from 2013 to 2020. The difference between the first and the second scenario was the change in the number of deaths exclusively attributable to population ageing. The difference between the second scenario and the number of deaths observed in 2020 was the change in the number of deaths exclusively attributable to age-specific mortality rate.

## Results

### Expected Deaths and the Mortality Rate of Lymphoma in 2020

There were an estimated 31,225 deaths due to lymphoma, with a crude mortality rate of 2.26 per 100,000 population. The ASMRC and ASMRW per 100,000 population were 1.76 and 1.35, respectively (**Table 1**). For HL, the number of deaths was 1,838, with crude mortality rate, ASMRC and ASMRW of 0.13, 0.10 and 0.08 per 100,000 population, respectively. For NHL, the number of deaths was 29,387, with crude mortality rate, ASMRC and ASMRW of 2.13, 1.66 and 1.27 per 100,000 population, respectively.

**Table 1:** Mortality rate of lymphoma by sex and residence in China, 2020.

	Sex	Crude rate(1/10^5^)	ASMRC(1/10^5^)	ASMRW(1/10^5^)
Lymphoma				
All	Both	2.26	1.76	1.35
	Male	2.73	2.26	1.73
	Female	1.78	1.30	0.98
Urban	Both	2.20	1.72	1.30
	Male	2.63	2.21	1.68
	Female	1.76	1.27	0.95
Rural	Both	2.30	1.79	1.38
	Male	2.79	2.30	1.77
	Female	1.79	1.31	1.00
Hodgkin lymphoma				
All	Both	0.13	0.10	0.08
	Male	0.16	0.14	0.10
	Female	0.10	0.08	0.06
Urban	Both	0.12	0.09	0.07
	Male	0.14	0.12	0.09
	Female	0.09	0.07	0.06
Rural	Both	0.14	0.11	0.09
	Male	0.18	0.15	0.11
	Female	0.11	0.08	0.06
Non-Hodgkin lymphoma				
All	Both	2.13	1.66	1.27
	Male	2.56	2.12	1.63
	Female	1.68	1.22	0.92
Urban	Both	2.09	1.62	1.23
	Male	2.49	2.08	1.59
	Female	1.67	1.20	0.90
Rural	Both	2.16	1.68	1.29
	Male	2.61	2.15	1.66
	Female	1.69	1.24	0.94

ASMRC, age-standardized mortality rate adjusted by the Chinese standard population; ASMRW, age-standardized mortality rate adjusted by the world standard population.

### Mortality Rates of Lymphoma Stratified by Age and Sex in 2020

**Figure 1:**
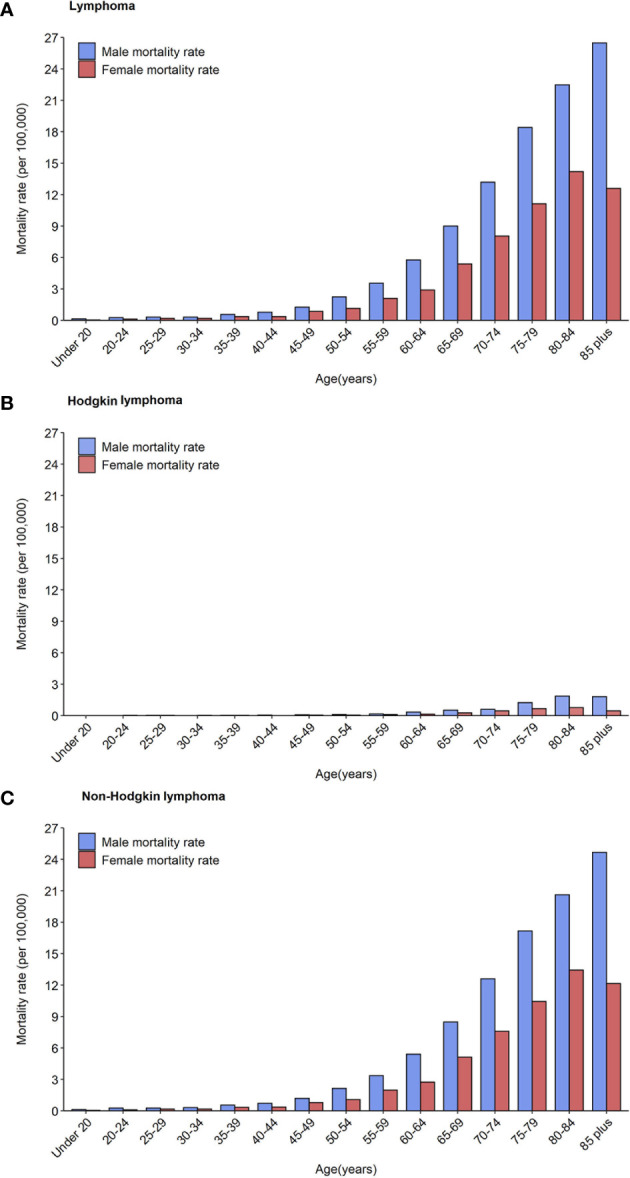
Mortality rates of lymphoma **(A)**, Hodgkin lymphoma **(B)** and non-Hodgkin lymphoma **(C)** by age groups and sex in 2020.

In total, the age-specific mortality rate of lymphoma increased with age and reached a peak (18.04 per 100,000 population) in the age group of over 85 years (**Figure 1A**; **Table 2**). An upward trend in mortality rate with age was observed in both HL and NHL. The peak mortality rate was observed in the age group of 80–84 years for HL and over 85 years for NHL.

**Table 2:** Mortality rate of lymphoma stratified by age, sex, region and residence, 2020.

Age groups	Gender				Residence			Region		
	Both	Male	Female		Urban	Rural		Eastern	Central	Western
Lymphoma										
0~	0.10	0.15	0.05		0.10	0.11		0.09	0.10	0.13
20~	0.21	0.28	0.14		0.22	0.21		0.27	0.12	0.22
25~	0.26	0.32	0.20		0.15	0.37		0.23	0.28	0.30
30~	0.25	0.31	0.19		0.16	0.34		0.22	0.26	0.32
35~	0.47	0.58	0.36		0.42	0.52		0.49	0.36	0.55
40~	0.59	0.79	0.37		0.47	0.68		0.64	0.63	0.44
45~	1.08	1.28	0.87		0.86	1.24		1.09	1.04	1.10
50~	1.71	2.26	1.14		1.19	2.08		1.59	1.88	1.70
55~	2.83	3.55	2.10		2.48	3.09		2.95	2.85	2.55
60~	4.35	5.78	2.90		4.00	4.58		4.80	4.47	3.36
65~	7.17	9.02	5.40		7.02	7.26		7.72	7.43	5.78
70~	10.55	13.20	8.06		10.33	10.70		12.38	9.79	8.18
75~	14.54	18.42	11.13		15.85	13.60		16.78	14.14	10.81
80~	17.81	22.47	14.20		21.01	15.47		19.32	16.60	15.95
85~	18.04	26.49	12.61		23.75	13.85		22.14	13.95	14.04
Hodgkin lymphoma										
0~	0.01	0.01	0.01		0.00	0.01		0.00	0.01	0.02
20~	0.02	0.01	0.02		0.04	0.00		0.03	0.00	0.02
25~	0.04	0.04	0.03		0.02	0.05		0.03	0.03	0.04
30~	0.01	0.00	0.03		0.00	0.03		0.01	0.03	0.00
35~	0.03	0.03	0.03		0.04	0.02		0.02	0.05	0.02
40~	0.03	0.06	0.00		0.02	0.04		0.03	0.03	0.03
45~	0.08	0.09	0.07		0.05	0.09		0.06	0.09	0.09
50~	0.09	0.10	0.07		0.05	0.12		0.06	0.12	0.10
55~	0.15	0.19	0.11		0.09	0.20		0.12	0.21	0.13
60~	0.25	0.35	0.15		0.25	0.25		0.24	0.25	0.27
65~	0.39	0.53	0.26		0.36	0.42		0.36	0.43	0.42
70~	0.53	0.61	0.46		0.55	0.52		0.57	0.57	0.41
75~	0.95	1.25	0.68		0.97	0.93		0.95	1.01	0.86
80~	1.24	1.86	0.77		1.31	1.20		1.16	1.72	0.80
85~	0.99	1.81	0.47		0.84	1.10		1.26	0.90	0.51
Non-Hodgkin lymphoma										
0~	0.10	0.14	0.05		0.10	0.10		0.09	0.09	0.12
20~	0.20	0.27	0.12		0.18	0.21		0.25	0.12	0.20
25~	0.23	0.28	0.17		0.13	0.32		0.20	0.25	0.26
30~	0.24	0.31	0.17		0.16	0.31		0.21	0.23	0.32
35~	0.44	0.55	0.33		0.38	0.50		0.47	0.31	0.53
40~	0.55	0.73	0.37		0.45	0.64		0.61	0.60	0.41
45~	1.00	1.20	0.80		0.81	1.14		1.03	0.96	1.01
50~	1.62	2.15	1.07		1.15	1.96		1.54	1.76	1.60
55~	2.68	3.37	1.99		2.39	2.90		2.83	2.64	2.42
60~	4.09	5.42	2.75		3.75	4.33		4.55	4.21	3.09
65~	6.77	8.49	5.13		6.67	6.84		7.36	7.01	5.36
70~	10.02	12.59	7.60		9.78	10.18		11.81	9.21	7.77
75~	13.59	17.17	10.45		14.88	12.67		15.83	13.13	9.95
80~	16.57	20.62	13.44		19.71	14.27		18.15	14.88	15.15
85~	17.04	24.68	12.14		22.91	12.74		20.88	13.05	13.52

The risk of death due to lymphoma was approximately 1.5–2 times greater in males than in females in all age groups. For HL, the age-specific mortality rate was less than one per 100,000 population in those younger than 75 years in males, and in all age groups in females (**Figure 1B**). For NHL, the age-specific mortality rate gradually increased and reached a maximum in males over 85 years (24.68 per 100,000 population) and females aged 80–84 years (13.44 per 100,000 population, **Figure 1C**).

### Mortality Rates of Lymphoma Stratified by Regions and Provinces in 2020

**Figure 2:**
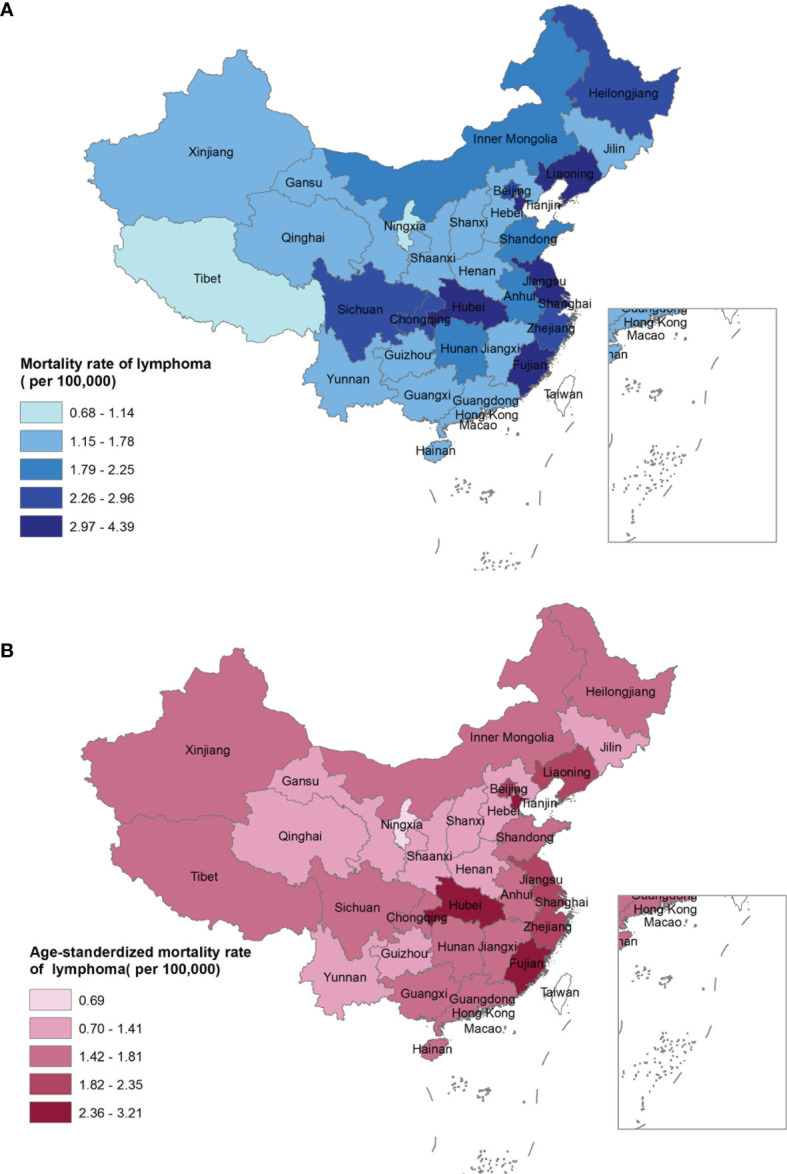
Crude mortality rate **(A)** and age-standardized mortality rate of China **(B)** of lymphoma for both sexes by provinces in 2020, China.

In total, a heterogeneous distribution of mortality rates was observed (**Appendix Figures S1**, **S2**). Eastern China had a higher crude mortality rate and ASMRC than central and western China (**Appendix Table S1**; **Figures 2A, B**).

At the provincial level, the crude mortality rate was highest in Hubei (4.39 per 100,000 population), Tianjin (3.86 per 100,000 population), and Liaoning (3.53 per 100,000 population), while was lowest in Ningxia (0.68 per 100,000 population), Tibet (1.14 per 100,000 population), and Qinghai (1.29 per 100,000 population) (**Figure 2A**). In contrast, the highest ASMRC was observed in Hubei (3.21 per 100,000 population), Fujian (3.17 per 100,000 population), and Tianjin (2.77 per 100,000 population), while the lowest ASMRC was seen in Ningxia (0.69 per 100,000 population), Hebei (1.04 per 100,000 population), and Jilin (1.15 per 100,000 population, **Figure 2B**).

### Trends in Mortality of Lymphoma From 2013 to 2020

The mortality rate decreased by 1.85% during 2013–2020 (−22.94% for HL and −0.14% for NHL, **Table 3**). The change was attributed to three factors: population growth (5.13%), population aging (21.57%), and age-specific mortality rate (−28.55%, **Table 4**).

**Table 3:** Mortality rate and average annual percentage change of lymphoma by sex in China, 2013-2020.

	Mortality ratein 2013	Mortality ratein 2020	AAPC(95% CI, %)	*P* value
Lymphoma				
Male				
Crude rate (1/10^5^)	3.05	2.73	-0.6 (-2.5 to 1.3)	0.458
ASMRC (1/10^5^)	3.03	2.26	-3.1 (-5.1 to -1.1)	0.009
ASMRW (1/10^5^)	2.36	1.73	-4.0 (-6.7 to -1.2)	0.005
Female				
Crude rate (1/10^5^)	1.77	1.78	0.3 (-1.8 to 2.5)	0.737
ASMRC (1/10^5^)	1.61	1.30	-3.3 (-6.3 to -0.3)	0.034
ASMRW (1/10^5^)	1.27	0.98	-3.9 (-6.8 to -0.8)	0.014
Hodgkin lymphoma				
Male				
Crude rate (1/10^5^)	0.23	0.16	-4.7 (-7.4 to -1.8)	0.007
ASMRC (1/10^5^)	0.23	0.14	-7.1 (-9.6 to -4.5)	0.001
ASMRW (1/10^5^)	0.18	0.10	-7.6 (-9.9 to -5.2)	< 0.001
Female				
Crude rate (1/10^5^)	0.13	0.10	-4.0 (-7.2 to -0.8)	0.023
ASMRC (1/10^5^)	0.12	0.08	-6.7 (-9.6 to -3.7)	0.002
ASMRW (1/10^5^)	0.10	0.06	-6.9 (-9.4 to -4.3)	0.001
Non-Hodgkin lymphoma				
Male				
Crude rate (1/10^5^)	2.82	2.56	-0.3 (-2.3 to 1.7)	0.724
ASMRC (1/10^5^)	2.80	2.12	-3.4 (-6.3 to -0.4)	0.026
ASMRW (1/10^5^)	2.18	1.63	-3.7 (-6.6 to -0.7)	0.015
Female				
Crude rate (1/10^5^)	1.64	1.68	0.6 (-1.6 to 2.9)	0.517
ASMRC (1/10^5^)	1.49	1.22	-2.6 (-5.9 to 0.8)	0.131
ASMRW (1/10^5^)	1.17	0.92	-3.6 (-6.6 to -0.6)	0.019

AAPC, average annual percentage change; CI, confidence interval

**Table 4:** Decomposition of changes in lymphoma deaths from 2013 to 2020.

	Lymphoma	Hodgkin lymphoma	Non-Hodgkin lymphoma

Both
Observed number of people in 2013	7,813*	586*	7,227*
Number expected with 2020 population, 2013 population age structure, and 2013 deaths	8,213	616	7,597
Number expected with 2020 population, 2020 population age structure, and 2013 deaths	9,898	746	9,151
Observed number of people in 2020	7,667*	451*	7,216*
Percentage change from 2013 due to population growth	5.13	5.13	5.13
Percentage change from 2013 due to population ageing	21.57	22.32	21.51
Percentage change from 2013 due to change in age-specific mortality rate	-28.55	-50.39	-26.78
Observed percentage change from 2013 to 2020	-1.85	-22.94	-0.14
Male			
Observed number of people in 2013	5,032*	382*	4,650*
Number expected with 2020 population, 2013 population age structure, and 2013 deaths	5,271	400	4,871
Number expected with 2020 population, 2020 population age structure, and 2013 deaths	6,297	483	5,813
Observed number of people in 2020	4,713*	285*	4,428*
Percentage change from 2013 due to population growth	4.73	4.73	4.73
Percentage change from 2013 due to population ageing	20.40	21.87	20.28
Percentage change from 2013 due to change in age-specific mortality rate	-31.49	-52.08	-29.80
Observed percentage change from 2013 to 2020	-6.36	-25.48	-4.79
Female			
Observed number of people in 2013	2,780*	204*	2,576*
Number expected with 2020 population, 2013 population age structure, and 2013 deaths	2,933	215	2,718
Number expected with 2020 population, 2020 population age structure, and 2013 deaths	3,567	260	3,308
Observed number of people in 2020	2,955*	167*	2,788*
Percentage change from 2013 due to population growth	5.54	5.54	5.54
Percentage change from 2013 due to population ageing	22.81	21.98	22.88
Percentage change from 2013 due to change in age-specific mortality rate	-22.03	-45.65	-20.16
Observed percentage change from 2013 to 2020	6.33	-18.13	8.26

*Death number was based on the disease surveillance points system of Chinese Center for Disease Control and Prevention.

**Figure 3:**
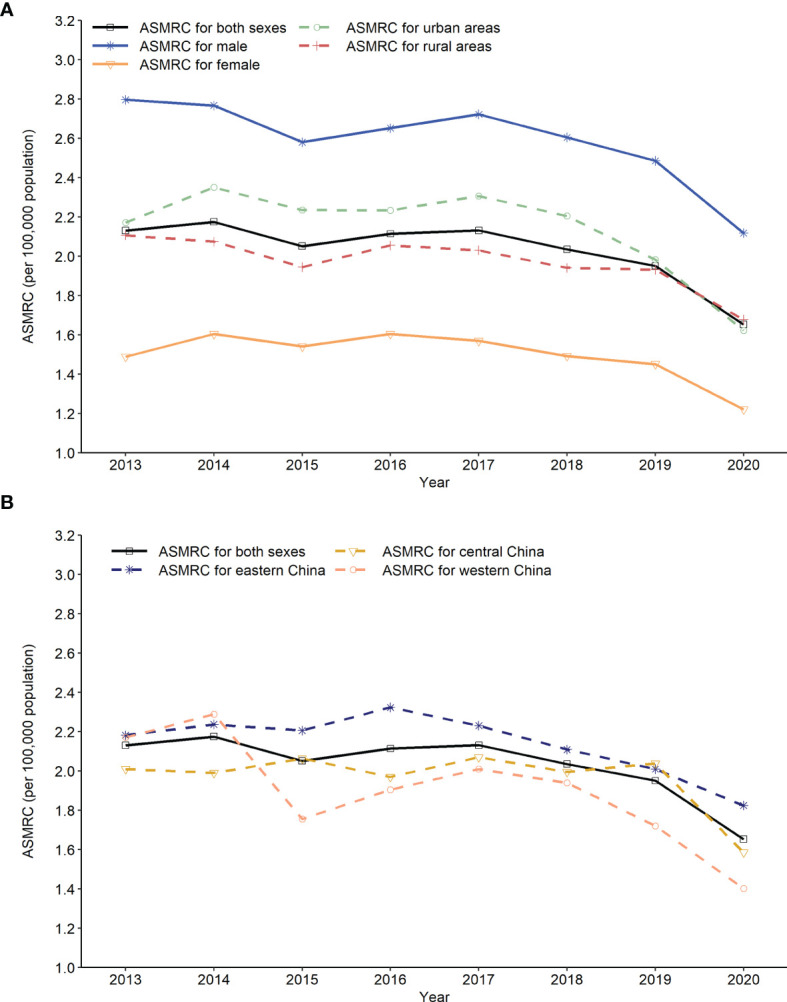
Trends in age-standardized mortality rate of China (ASMRC) of lymphoma by sex, residence and region in China, 2013 to 2020. **(A)** Trends in ASMRC of lymphoma by gender and residence, **(B)** Trends in ASMRC of lymphoma by region.

The ASMRC per 100,000 population decreased from 2.30 in 2013 to 1.76 in 2020, which resulted in an AAPC of −3.6% (95% CI: −5.6% to −1.5%). In terms of residence variation, the ASMRC showed a decrease of 0.66 per 100,000 population with an AAPC of −4.7% in urban areas, and a decrease of 0.47 per 100,000 population with an AAPC of −2.5% in rural areas (**Appendix Table S2**, **Figure 3A**). All regions including eastern, central and western China showed a downward trend in the ASMRC (**Appendix Table S3**; **Figure 3B**). Moreover, the ASMRC of both HL and NHL in all areas showed a significant downward trend (**Table 3**).

## Discussion

The present study is the most comprehensive evaluation of lymphoma mortality based on the CDC-DSP system. The 605 surveillance points in this system were selected using an iterative method of multistage stratification and had a good national and regional representativeness at both national and provincial levels (10). We determined the temporal downward trend in lymphoma mortality during the past decade. Moreover, we explored the demographic and geographical differences in lymphoma mortality.

Older individuals often have a higher cancer burden. For example, the cancer burden was lowest in those aged 35–39 years and highest in those aged 80–84 years in China (11). Similarly, the probability of developing NHL in those older than 70 years was 6–7 fold higher than that in those younger than 60 years, which was 3–4 fold higher than that in the age group of 60–69 years in the United States (12). Moreover, NHL contributed to 4% of cancer deaths in males and 5% of cancer deaths in females aged over 85 years in 2019 (13). Consistent with previous studies, the mortality of lymphoma showed an upward trend with age and reached a peak in the age group of over 85 years in the present study. Notably, the mortality rate was very low (less than one per 100,000 population) in the age groups of < 45 years and was very high (more than 10 per 100,000 population) in the age groups of > 70 years. This phenomenon may be partly explained by a higher age-specific incidence rate, poor chemotherapy tolerance, complications due to therapy, and lower survival rates in the elderly (14–17). These findings highlight the need to develop differentiated disease control and prevention strategies for different age-specific populations.

The level of economic development played an important role in the heterogeneous geographical distribution of the cancer burden. A cohort study involving 497,693 participants aged between 35 and 74 years showed that the standardized mortality rates were higher in rural areas (241.2 per 100,000 person-years) than in urban areas (183.5 per 100,000 person-years) (18). Moreover, the cancer mortality rate was 44% higher in rural men aged 30–34 years and 44% higher in rural women aged 15–19 years than in their urban counterparts (19). In the present study, rural areas had a higher mortality rate of lymphoma than the urban areas, especially with a 22% higher mortality rate of HL. However, the incidence rates of lymphoid neoplasms in rural areas were lower than those in urban areas (3.4 vs 4.7 per 100,000 population) in China (20). This urban–rural discordance may be partly explained by an increase in deaths due to insufficient access to health services and poor survival in rural areas (21–24). Therefore, these findings support the establishment of an official medical system to reduce the burden of lymphoma, especially in rural areas.

Importantly, there was a significant decrease in lymphoma mortality during 2013–2020. In particular, despite the increased incidence due to population growth and aging, the HL mortality rate decreased by about a quarter, due to a 50% reduction in the age-specific mortality rates. This was associated with a better prognosis due to advances in anti-lymphoma treatment (25, 26). For example, a study involving 3,760 lymphoma patients showed that the 5- and 10-year overall survival rates for classic HL were 80% and 71%, respectively, and the 5-year overall survival rate for classic HL increased by 25% during the past two decades (55.4% in 1996–2000 vs 79.0% in 2010–2015) (27). Similarly, immunochemotherapy with rituximab improved the prognosis of B-cell lymphoma. For example, the introduction of rituximab into therapy for diffuse large B-cell lymphoma led to better survival outcomes compared to chemotherapy alone (28–30). However, the 5-year relative survival rate of lymphoid malignancies was only 38.3% in China, which was markedly lower than that in the western countries (70% or higher) (31). Careful attention should be paid to the increase in the mortality rate of both HL and NHL due to the population in China. Therefore, further studies should focus on evaluating the impact of the promotion of standardized diagnosis and treatment procedures on lymphoma burden in the absence of prevention measures.

This study has several limitations. First, the data were extracted according to the International Classification of Diseases- 10 codes from the CDC-DSP database, the mortality rates of lymphoma subtypes were not assessed according to the lymphoid tumor classifications of the World Health Organization. Second, the mortality rate of HL was very low (< 0.1 per 100,000 population) in the age groups less than 55 years at the national level, which may lead to underestimation or overestimation of the results at the provincial level. Third, socioeconomic factors such as sociodemographic index and human development index, were not used to evaluate the attribution to the change in disease burden.

In conclusion, the present study determined the spatiotemporal characteristics of lymphoma mortality using nationally representative data from China. The mortality rate was higher in males and older individuals. Moreover, rural areas had higher mortality rates than urban areas. An encouraging downward trend was observed, especially in HL mortality. Moreover, the present study provided detailed information on the mortality rate of lymphoma at the national and provincial levels. These results may assist in establishing stratified strategies when policies for disease prevention and management are implemented.

## Data Availability Statement

The original contributions presented in the study are included in the article/**Supplementary Material**. Further inquiries can be directed to the corresponding authors.

## Author Contributions

WL conceived and designed the study, analyzed the data, and drafted and revised the paper. JQ and JL prepared and analyzed the data. YS and LW drafted and revised the manuscript. MZ, JM, and JZ designed the study, interpreted the results, and drafted and revised the paper. All the authors have provided critical comments on the manuscript.

## Funding

This study was supported by the Clinical Research Fund for Distinguished Young Scholars of Beijing Cancer Hospital (grant no. QNJJ202106). The funders of the study had no role in study design, data collection, data analysis, data interpretation, or writing of the report.

## Conflict of Interest

The authors declare that the research was conducted in the absence of any commercial or financial relationships that could be construed as a potential conflict of interest.

## Publisher’s Note

All claims expressed in this article are solely those of the authors and do not necessarily represent those of their affiliated organizations, or those of the publisher, the editors and the reviewers. Any product that may be evaluated in this article, or claim that may be made by its manufacturer, is not guaranteed or endorsed by the publisher.

## Supplementary Material

Supplementary Figure 1 Mortality rate of lymphoma in males by provinces in 2020, China**. (A**) Crude mortality rate; **(B**) age-standardized mortality rate of China.

Supplementary Figure 2 Mortality rate of lymphoma in females by provinces in 2020, China**. (A**) Crude mortality rate; **(B**) age-standardized mortality rate of China.
