# Supplementary material for: Mortality Rate of Lymphoma in China, 2013–2020
Source: Front Oncol. 2022 Jun 7;12:902643. doi: 10.3389/fonc.2022.902643 (PMC9209711; doi:10.3389/fonc.2022.902643)
Supplement: Supplementary file 3 [file Table_1.docx]

Table S1 Mortality rate of lymphoma by sex and region in China, 2020

|  | Sex | Crude rate | ASMRC | ASMRW |
| --- | --- | --- | --- | --- |
|  |  | (1/10^5^） | (1/10^5^） | (1/10^5^） |
| Lymphoma |  |  |  |  |
| Eastern China | Both | 2.56 | 1.92 | 1.47 |
|  | Male | 3.08 | 2.50 | 1.91 |
|  | Female | 2.01 | 1.40 | 1.06 |
| Central China | Both | 2.21 | 1.70 | 1.31 |
|  | Male | 2.70 | 2.21 | 1.69 |
|  | Female | 1.69 | 1.23 | 0.95 |
| Western China | Both | 1.80 | 1.50 | 1.15 |
|  | Male | 2.12 | 1.85 | 1.44 |
|  | Female | 1.47 | 1.16 | 0.88 |
| Hodgkin lymphoma |  |  |  |  |
| Eastern China | Both | 0.13 | 0.10 | 0.08 |
|  | Male | 0.17 | 0.14 | 0.10 |
|  | Female | 0.09 | 0.07 | 0.05 |
| Central China | Both | 0.15 | 0.12 | 0.09 |
|  | Male | 0.18 | 0.15 | 0.11 |
|  | Female | 0.13 | 0.10 | 0.07 |
| Western China | Both | 0.11 | 0.10 | 0.08 |
|  | Male | 0.14 | 0.12 | 0.10 |
|  | Female | 0.09 | 0.07 | 0.05 |
| Non-Hodgkin lymphoma |  |  |  |  |
| Eastern China | Both | 2.43 | 1.82 | 1.39 |
|  | Male | 2.91 | 2.36 | 1.81 |
|  | Female | 1.92 | 1.33 | 1.00 |
| Central China | Both | 2.05 | 1.58 | 1.22 |
|  | Male | 2.53 | 2.06 | 1.59 |
|  | Female | 1.57 | 1.14 | 0.87 |
| Western China | Both | 1.68 | 1.40 | 1.08 |
|  | Male | 1.98 | 1.72 | 1.34 |
|  | Female | 1.38 | 1.09 | 0.83 |

ASMRC，age-standardized mortality rate adjusted by the Chinese standard population; ASMRW, age-standardized mortality rate adjusted by the world standard population.
