# Supplementary material for: Mortality Rate of Lymphoma in China, 2013–2020
Source: Front Oncol. 2022 Jun 7;12:902643. doi: 10.3389/fonc.2022.902643 (PMC9209711; doi:10.3389/fonc.2022.902643)
Supplement: Supplementary file 4 [file Table_2.docx]

Table S2 Change of mortality rate of lymphoma by residence in China, 2013-2020

|  | Mortality rate  in 2013 | Mortality rate  in 2020 | AAPC  (95% CI, %) | *P* value |
| --- | --- | --- | --- | --- |
| Lymphoma |  |  |  |  |
| Urban |  |  |  |  |
| Crude rate (1/10^5^) | 2.50 | 2.20 | -1.6  (-4 to 0.8) | 0.196 |
| ASMRC (1/10^5^) | 2.37 | 1.72 | -4.7  (-7.5 to -1.8) | 0.002 |
| ASMRW (1/10^5^) | 1.83 | 1.30 | -5  (-7.8 to -2.2) | 0.001 |
| Rural |  |  |  |  |
| Crude rate (1/10^5^) | 2.38 | 2.30 | 0.2  (-1.5 to 2.0) | 0.785 |
| ASMRC (1/10^5^) | 2.26 | 1.79 | -2.5  (-4.2 to -0.8) | 0.012 |
| ASMRW (1/10^5^) | 1.79 | 1.38 | -2.8  (-4.9 to -0.8) | 0.015 |
| Hodgkin lymphoma |  |  |  |  |
| Urban |  |  |  |  |
| Crude rate (1/10^5^) | 0.21 | 0.12 | -8  (-11.4 to -4.5) | <0.001 |
| ASMRC (1/10^5^) | 0.20 | 0.09 | -9.9  (-13.1 to -6.5) | <0.001 |
| ASMRW (1/10^5^) | 0.15 | 0.07 | -10.2  (-12.4 to -8.1) | <0.001 |
| Rural |  |  |  |  |
| Crude rate (1/10^5^) | 0.16 | 0.14 | -2.3  (-5.8 to 1.4) | 0.175 |
| ASMRC (1/10^5^) | 0.16 | 0.11 | -5.1  (-8.3 to -1.7) | 0.011 |
| ASMRW (1/10^5^) | 0.13 | 0.09 | -5.6  (-8.9 to -2.2) | 0.007 |
| Non-Hodgkin lymphoma |  |  |  |  |
| Urban |  |  |  |  |
| Crude rate (1/10^5^) | 2.29 | 2.09 | -1.2  (-3.6 to 1.3) | 0.334 |
| ASMRC (1/10^5^) | 2.17 | 1.62 | -4.3  (-7.2 to -1.4) | 0.004 |
| ASMRW (1/10^5^) | 1.68 | 1.23 | -4.7  (-7.7 to -1.6) | 0.003 |
| Rural |  |  |  |  |
| Crude rate (1/10^5^) | 2.21 | 2.16 | 0.4  (-1.3 to 2.2) | 0.600 |
| ASMRC (1/10^5^) | 2.11 | 1.68 | -2.3  (-4 to -0.6) | 0.018 |
| ASMRW (1/10^5^) | 1.67 | 1.29 | -2.6  (-4.7 to -0.5) | 0.022 |

AAPC, average annual percentage change; CI, confidence interval
