# Supplementary material for: Mortality Rate of Lymphoma in China, 2013–2020
Source: Front Oncol. 2022 Jun 7;12:902643. doi: 10.3389/fonc.2022.902643 (PMC9209711; doi:10.3389/fonc.2022.902643)
Supplement: Supplementary file 5 [file Table_3.docx]

Table S3 Change of mortality rate of lymphoma by region in China, 2013-2020

|  | Mortality rate  in 2013 | Mortality rate  in 2020 | AAPC  (95% CI, %) | *P* value |
| --- | --- | --- | --- | --- |
| Lymphoma |  |  |  |  |
| Eastern |  |  |  |  |
| Crude rate (1/10^5^) | 2.60 | 2.56 | 0  (-1.5 to 1.6) | 0.991 |
| ASMRC (1/10^5^) | 2.37 | 1.92 | -2.6  (-4.5 to -0.6) | 0.010 |
| ASMRW (1/10^5^) | 1.85 | 1.47 | -3.2  (-4.9 to -1.5) | <0.001 |
| Central |  |  |  |  |
| Crude rate (1/10^5^) | 2.25 | 2.21 | 0.9  (-1.5 to 3.4) | 0.381 |
| ASMRC (1/10^5^) | 2.18 | 1.70 | -2.2  (-4.9 to 0.6) | 0.104 |
| ASMRW (1/10^5^) | 1.72 | 1.31 | -3.4  (-5.9 to -0.7) | 0.013 |
| Western |  |  |  |  |
| Crude rate (1/10^5^) | 2.33 | 1.80 | -2.6  (-5.8 to 0.8) | 0.108 |
| ASMRC (1/10^5^) | 2.32 | 1.50 | -4.9  (-8.3 to -1.3) | 0.017 |
| ASMRW (1/10^5^) | 1.83 | 1.15 | -5.1  (-8.9 to -1) | 0.023 |
| Hodgkin lymphoma |  |  |  |  |
| Eastern |  |  |  |  |
| Crude rate (1/10^5^) | 0.21 | 0.13 | -6.0  (-10.6 to -1.1) | 0.024 |
| ASMRC (1/10^5^) | 0.19 | 0.10 | -8.4  (-12.9 to -3.8) | 0.005 |
| ASMRW (1/10^5^) | 0.15 | 0.08 | -8.7  (-13.4 to -3.8) | 0.005 |
| Central |  |  |  |  |
| Crude rate (1/10^5^) | 0.17 | 0.15 | -3.2  (-6.2 to -0.1) | 0.047 |
| ASMRC (1/10^5^) | 0.17 | 0.12 | -6.2  (-9.4 to -2.9) | 0.004 |
| ASMRW (1/10^5^) | 0.13 | 0.09 | -6.8  (-9.9 to -3.5) | 0.002 |
| Western |  |  |  |  |
| Crude rate (1/10^5^) | 0.15 | 0.11 | -3.3  (-6.8 to 0.3) | 0.064 |
| ASMRC (1/10^5^) | 0.15 | 0.10 | -5.8  (-8.4 to -3.1) | 0.002 |
| ASMRW (1/10^5^) | 0.12 | 0.08 | -6.1  (-8.8 to -3.3) | 0.002 |
| Non-Hodgkin lymphoma |  |  |  |  |
| Eastern |  |  |  |  |
| Crude rate (1/10^5^) | 2.40 | 2.43 | 0.6  (-0.7 to 1.9) | 0.378 |
| ASMRC (1/10^5^) | 2.18 | 1.82 | -2.5  (-4.0 to -1.0) | 0.001 |
| ASMRW (1/10^5^) | 1.70 | 1.39 | -2.8  (-4.2 to -1.4) | <0.001 |
| Central |  |  |  |  |
| Crude rate (1/10^5^) | 2.07 | 2.05 | 1.2  (-1.4 to 3.9) | 0.291 |
| ASMRC (1/10^5^) | 2.01 | 1.58 | -1.9  (-4.8 to 1.1) | 0.176 |
| ASMRW (1/10^5^) | 1.59 | 1.22 | -2.3  (-5.5 to 1.1) | 0.146 |
| Western |  |  |  |  |
| Crude rate (1/10^5^) | 2.18 | 1.68 | -2.5  (-5.8 to 0.9) | 0.121 |
| ASMRC (1/10^5^) | 2.17 | 1.40 | -4.8  (-8.4 to -1.1) | 0.02 |
| ASMRW (1/10^5^) | 1.71 | 1.08 | -5.0  (-9.0 to -0.8) | 0.027 |

AAPC, average annual percentage change; CI, confidence interval
